# Kounis Syndrome: An Allergic Acute Coronary Syndrome Due to a Bee Sting

**DOI:** 10.7759/cureus.26395

**Published:** 2022-06-28

**Authors:** Ei Ei Thwe, Palina Sudnik, Carmen Dobrovolschi, Mahesh Krishnamurthy

**Affiliations:** 1 Internal Medicine, St. Luke's Hospital, Bethlehem, USA; 2 Internal Medicine, St. Luke's University Health Network, Bethlehem, USA; 3 Internal Medicine, St. Luke’s University Health Network, Bethlehem, USA

**Keywords:** st elevations, allergic reaction, bee sting, mi kounis, allergic acute coronary syndrome

## Abstract

Kounis syndrome, also known as allergic acute coronary syndrome, is defined as the occurrence of acute coronary syndrome (ACS) in the setting of an allergic or hypersensitivity reaction. Although Kounis syndrome is not an uncommon disease, many cases go undiagnosed or unrecognized. Patients with systemic allergic reactions associated with evidence of ACS should be suspected of Kounis syndrome because the outcome could be devastating if not treated promptly. The physician must be aware of Kounis syndrome because the treatment modality differs from traditional ACS.

## Introduction

The concept of allergic angina was introduced by Kounis and Zavras in 1991 [1]. Allergic angina, also known as Kounis syndrome, is believed to be caused by the release of inflammatory mediators such as histamine, platelet-activating factor, leukotrienes, neutral proteases, and a variety of cytokines and chemokines during an allergic reaction. These mediators have the potential to trigger either coronary artery spasm or an immediate coronary thrombosis that may lead to acute myocardial injury [1-3].

Patients with systemic allergic reactions associated with evidence of acute coronary syndrome (ACS) should be suspected of Kounis syndrome because of its potential life-threatening nature. Kounis syndrome is not an uncommon disease [4]. However, it is believed that a significant number of cases go undiagnosed or unrecognized [4]. Here, we would like to discuss the case of Kounis syndrome, which was caused by a bee sting.

## Case presentation

**Figure 1:**
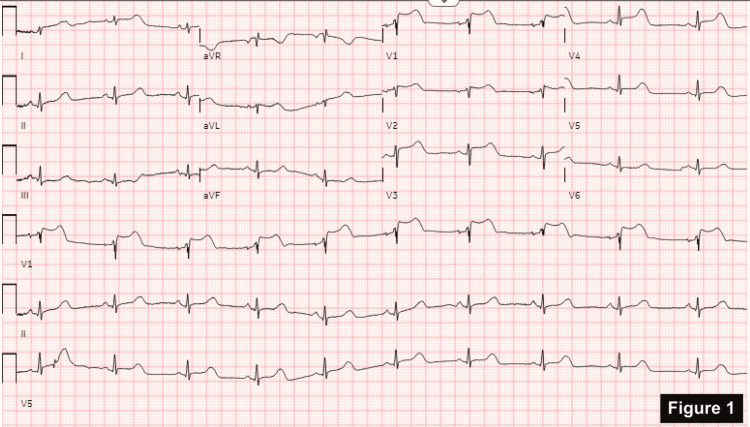
ST-segment elevation in V1-V4.

A relatively healthy 57-year-old female with no significant cardiac history presented to the emergency department one hour after being stung by a bee on her ankle. She complained of generalized urticaria, difficulty breathing, and tightness in her chest and throat. She did not recall any history of bee stings or anaphylaxis in the past. She denies any environmental or drug allergies. Her initial vital signs showed significant hypotension (68/39 mmHg). A stat electrocardiogram (ECG) showed ST-segment elevation in V1-V4 (Figure 1). Troponin was negative. Bedside cardiac ultrasound in the emergency room showed normal wall motion with preserved left ventricular function.

**Figure 2:**
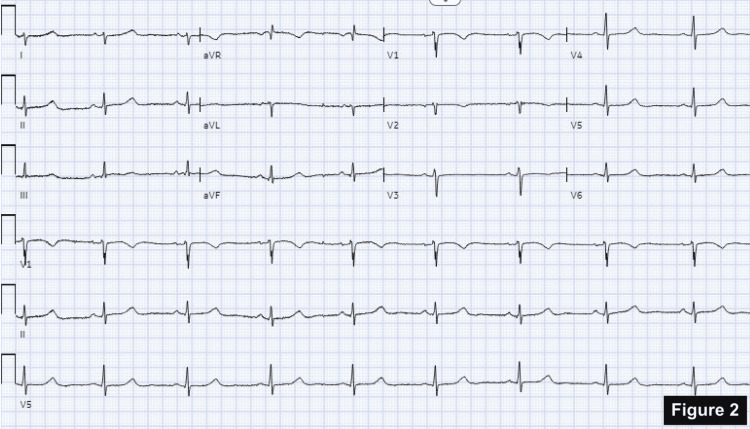
Normalization of ST-segment elevations.

She was treated with antihistamine and steroids for anaphylaxis. This resulted in the immediate normalization of her ST changes as well as an improvement in her blood pressure (Figure 2). She was not given an epinephrine injection due to concerns about coronary vasoconstriction, increased myocardial oxygen demand, and worsening myocardial ischemia.

She was admitted to the hospital for observation of her hemodynamic status as well as to monitor and treat any subsequent episodes. Laboratory studies, including complete blood count (showed no eosinophilia), basic metabolic panel, and serial troponins were unremarkable. Serial ECGs showed normal sinus rhythm with no significant ST-T wave changes. Formal transthoracic echocardiography revealed a normal ejection fraction with no regional wall motion abnormalities. She was diagnosed with Kounis syndrome, type 1 variant, due to transient ischemic ECG changes, which were completely resolved with the treatment of anaphylaxis. After 24 hours of observation, she was discharged home with an EpiPen, an antihistamine, a prescription for prednisone tapering, and a referral to cardiology and immunology.

## Discussion

Kounis syndrome affects patients of any age, and it can be caused by various triggers, including medications, intravenous contrast, food allergens, and environmental exposures. Presenting symptoms can be largely broad, ranging from minor symptoms such as flushing, nausea, vomiting, chest pain, and chest discomfort to major events such as hemodynamic instability and sudden death [2].

Three variants of Kounis syndrome have been described so far. Patients with type 1 variant have normal or nearly normal coronary arteries, and acute release of inflammatory mediators causes coronary vasospasm with or without elevation of cardiac enzymes. Type 2 variant affects patients with pre-existing atheromatous disease in whom mediator-induced vasospasm is accompanied by plaque erosion or rupture that manifests as an acute myocardial infarction. Type 3 variant is seen in patients with coronary artery stent thrombosis secondary to eosinophils and mast cell activation [3].

Effective Kounis syndrome management entails treating anaphylaxis while maintaining adequate myocardial perfusion [3,5]. The initial goal of treatment should be to prevent life-threatening anaphylaxis while keeping in mind that some medications may affect coronary blood flow. The drug of choice for anaphylaxis is epinephrine, but it can aggravate ischemia and worsen coronary vasospasm. Beta-blockers, on the other hand, can exaggerate coronary spasms due to the unopposed action of alpha-adrenergic receptors. According to Kounis, type 1 variant can be treated with corticosteroids and antihistamines alone [2]. A thorough cardiological work-up, including a 12-lead ECG, echocardiography, and cardiac risk factor modification, is required following the relief of the acute episode. Type 2 variant should be initially treated with the ACS protocol followed by corticosteroids and antihistamines. In addition to the ACS protocol, the type 3 variant necessitates urgent aspiration and histologic examination of the stent thrombus. Patients with allergic symptoms after stent implantation may benefit from antihistamines and corticosteroids. If allergic symptoms persisted despite medication, desensitization should be considered. If the above measures fail, stent removal would be the only option [2].

## Conclusions

Kounis syndrome is not a rare disease, however it is not well mentioned in the literature. It is important for physicians in clinical practice to recognize Kounis syndrome as it requires quick decision-making and prompt treatment.

## References

[1] Kounis NG, Zavras GM (1991). Histamine-induced coronary artery spasm: the concept of allergic angina. Br J Clin Pract.

[2] Kounis NG (2016). Kounis syndrome: an update on epidemiology, pathogenesis, diagnosis and therapeutic management. Clin Chem Lab Med.

[3] Haddad A, Smith T, Bole A, Shah M, Chakravarthy M (2018). Type I Kounis syndrome variant: a case report and literature review. Avicenna J Med.

[4] Li J, Zheng J, Zhou Y, Liu X, Peng W (2018). Acute coronary syndrome secondary to allergic coronary vasospasm (Kounis syndrome): a case series, follow-up and literature review. BMC Cardiovasc Disord.

[5] Sciatti E, Vizzardi E, Cani DS (2018). Kounis syndrome, a disease to know: case report and review of the literature. Monaldi Arch Chest Dis.

